# The docking protein Gab1 is the primary mediator of EGF-stimulated activation of the PI-3K/Akt cell survival pathway

**DOI:** 10.1186/1741-7007-2-24

**Published:** 2004-11-18

**Authors:** Dawn R Mattoon, Betty Lamothe, Irit Lax, Joseph Schlessinger

**Affiliations:** 1 Department of Pharmacology, Yale University School of Medicine, PO Box 208066, New Haven, CT 06520-8066, USA; 2 Current address: Protometrix, Inc./Invitrogen, 688 East Main Street, Branford, CT 06405, USA; 3 Current address: Department of Bioimmunotherapy, M.D. Anderson Cancer Center, 1515 Holcombe Blvd. Box 0143. Houston, TX 77030, USA

## Abstract

**Background:**

Gab1 is a docking protein that recruits phosphatidylinositol-3 kinase (PI-3 kinase) and other effector proteins in response to the activation of many receptor tyrosine kinases (RTKs). As the autophosphorylation sites on EGF-receptor (EGFR) do not include canonical PI-3 kinase binding sites, it is thought that EGF stimulation of PI-3 kinase and its downstream effector Akt is mediated by an indirect mechanism.

**Results:**

We used fibroblasts isolated from Gab1-/- mouse embryos to explore the mechanism of EGF stimulation of the PI-3 kinase/Akt anti-apoptotic cell signaling pathway. We demonstrate that Gab1 is essential for EGF stimulation of PI-3 kinase and Akt in these cells and that these responses are mediated by complex formation between p85, the regulatory subunit of PI-3 kinase, and three canonical tyrosine phosphorylation sites on Gab1. Furthermore, complex formation between Gab1 and the protein tyrosine phosphatase Shp2 negatively regulates Gab1 mediated PI-3 kinase and Akt activation following EGF-receptor stimulation. We also demonstrate that tyrosine phosphorylation of ErbB3 may lead to recruitment and activation of PI-3 kinase and Akt in Gab1-/- MEFs.

**Conclusions:**

The primary mechanism of EGF-induced stimulation of the PI-3 kinase/Akt anti-apoptotic pathway occurs via the docking protein Gab1. However, in cells expressing ErbB3, EGF and neuroregulin can stimulate PI-3 kinase and Akt activation in a Gab1-dependent or Gab1-independent manner.

## Background

Ligand stimulation of the epidermal growth factor receptor (EGFR) and the three other members of the EGFR family of receptor tyrosine kinases (ErbB2, ErbB3 and ErbB4) results in tyrosine autophosphorylation, recruitment of signaling proteins, and activation of distinct complement of signaling pathways that regulate a great variety of cellular responses [1,2].

One of the signaling pathways that is activated by the EGFR is the phosphatidylinositol-3 kinase (PI-3 kinase)/Akt anti-apoptic signaling pathway [3]. The PI-3 kinase holoenzyme consists of a regulatory subunit (p85) and a catalytic p110 subunit. The regulatory subunit contains two SH2 domains that bind specifically to pYXXM motifs in a variety of cellular proteins, including receptor tyrosine kinases such as the PDGF (platelet-derived growth factor) receptor, and c-kit and docking proteins such as IRS (insulin receptor substrate) 1, IRS2 and Gab1. Although the cytoplasmic domain of the EGFR does not contain any canonical p85 binding motifs, EGF stimulation leads to PI-3 kinase activation by an indirect mechanism. It has been proposed that the PI-3 kinase is activated in response to EGF stimulation as a consequence of formation of EGFR/ErbB3 heterodimers [4]. Unlike EGFR, the cytoplasmic region of ErbB3 contains at least six pYXXM motifs [5,6]. Indeed, EGF stimulation of cells co-expressing the EGFR and ErbB3 results in recruitment and activation of PI-3 kinase by tyrosine phosphorylated ErbB3 [4].

EGF stimulation of PI-3 kinase may also be mediated by the docking protein Gab1 (Grb2-associated binder-1). EGF stimulation leads to tyrosine phosphorylation of Gab1 enabling recruitment and activation of PI-3 kinase by the three canonical pYXXM motifs on Gab1 [7]. Gab1 was originally identified as a Grb2 binding protein, and was shown to be tyrosine phosphorylated in response to treatment with a variety of growth factors [7-9]. Gab1 contains a number of tyrosine residues that could serve as potential binding sites for the SH2 domain containing proteins Grb2, PI-3 kinase, and the protein tyrosine phosphatase Shp2 [10]. While there have been reports that Gab1 binds directly to the EGFR via an 83-amino acid stretch termed the Met-binding-domain or MBD [9], the majority of Gab1 is believed to be indirectly associated with the EGFR via the adaptor protein Grb2, which binds to a proline rich region on Gab1 via its C-terminal SH3 domain [10-13].

Cells over-expressing a mutant Gab1 protein containing tyrosine to phenylalanine mutations at the three p85 binding sites have been shown to be defective in EGF-induced JNK activation, and treatment of cells over-expressing wild type Gab1 with PI-3 kinase inhibitors interferes with MAPK signaling in response to EGF treatment, thus revealing a link between Gab1 and PI-3 kinase in EGF-induced mitogenic signaling [9,14]. Furthermore, the PI-3 kinase product phosphatidylinositol (3,4,5) triphosphate (PIP3) has been shown to bind to the pleckstrin homology (PH) domain of Gab1 resulting in membrane-association of Gab1, suggesting a positive feedback loop in which PI-3 kinase acts as both an upstream regulator and a downstream effector of Gab1 signaling via the EGFR [9]. Gab1 thus acts as a docking protein facilitating the recruitment of a multi-protein signaling complex including the EGFR, p85 and Shp2 in response to EGF treatment.

Although the role of the Shp2 protein in the control of EGFR/Gab1 interactions is not well understood, several studies have suggested that Gab1-associated Shp2 may influence EGF-induced PI-3 kinase signaling. Previous work has shown that Gab1 is not a global substrate of Shp2, as complex formation between Gab1 and Shp2 does not reduce the total EGF-induced tyrosine phosphorylation levels of Gab1 [15]. However there have been several reports suggesting that Shp2 may specifically de-phosphorylate the tyrosine phosphorylation sites on Gab1 that bind to p85, thus terminating recruitment of PI-3 kinase and EGF-induced activation of the PI-3 kinase pathway [16-18]. It has been shown that cells devoid of Shp2 show an increase in PI-3 kinase activity, as well as elevated and sustained levels of Akt activation in response to EGF treatment [18]. It was reported that treatment of cells with PI-3 kinase inhibitors or with the phosphatidylinositol (3,4,5) triphosphate (PIP3) phosphatase PTEN interferes with the association between Gab1 and Shp2 in response to EGF treatment, suggesting PI-3 kinase may be required for Shp2 recruitment of Gab1 following EGF stimulation [14]. However, the mechanism for this postulated recruitment is unknown.

In the experiments presented here we utilized fibroblasts isolated from Gab1-/- mouse embryos in order to examine the role of Gab1 in EGF-mediated activation of the PI-3 kinase/Akt cell survival pathway. We also address the question of whether ErbB3 recruitment of PI-3 kinase is dependent on or independent of Gab1. Our results demonstrate a clear requirement for Gab1 in recruitment and activation of PI-3 kinase in response to EGF stimulation. Additionally, we show that while Shp2 does not mediate global dephosphorylation of Gab1, it does appear to negatively regulate the EGF-induced activation of PI-3 kinase through an undefined mechanism. Finally we demonstrate that ErbB3 is capable of recruiting PI-3 kinase in the absence of Gab1, but Gab1 functions as the major mediator of PI-3 kinase activation in response to EGF stimulation.

## Results and Discussion

Previous studies have suggested that ErbB3 and Gab1 can function as links between EGFR and PI-3 kinase. In this report we use MEFs derived from Gab1 -/- embryos [19] to explore the contribution of Gab1 and ErbB3 to EGF stimulation of PI-3 kinase and Akt in these cells.

### Gab1 is essential for EGF stimulation of PI-3 kinase and Akt

**Figure 1:**
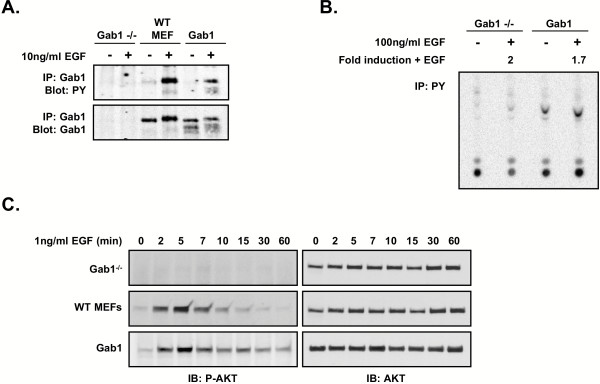
Expression of wild type Gab1 rescues EGF-induced PI-3 kinase and Akt activation in Gab1 deficient MEFs. **A. **The indicated cell lines were serum-starved for 24 hours and stimulated with 10 ng/ml EGF for five minutes at 37°C. Cell extracts were prepared and analyzed for Gab1 tyrosine phosphorylation and Gab1 expression. Both WT MEFs and cells expressing exogenous Gab1 show Gab1 tyrosine phosphorylation in response to EGF treatment. **B. **The indicated cell lines were serum-starved for 24 hours and stimulated with 100 ng/ml EGF for five minutes at 37°C. Cell extracts were prepared and phosphotyrosine immunoprecipitates were analyzed for PI-3 kinase activity. Gab1 -/- cells fail to show phosphotyrosine-associated PI-3 kinase activity, while cells expressing exogenous Gab1 display phosphotyrosine-associated PI-3 kinase activity that is augmented by EGF treatment. **C. **The indicated cell lines were serum-starved for 24 hours and stimulated with 1 ng/ml EGF for varying periods of time at 37°C. Cell extracts were prepared and analyzed for activation of Akt by using antibodies that specifically recognize the serine473 phosphorylated form of Akt. Membranes were subsequently stripped and immunoblotted for Akt to confirm equal loading. Ectopic expression of Gab1 in Gab1 -/- MEFs rescues the EGF-induced activation of Akt.

MEFs derived from Gab1-/- or wild type (WT) embryos were stimulated with EGF and assayed for Gab1 tyrosine phosphorylation, for activation of PI-3 kinase and for Akt stimulation. As shown in Figure 1A, the endogenous Gab1 present in WT MEFs is tyrosine phosphorylated in response to EGF treatment. As shown in Figure 1B, Gab1 -/- MEFs displayed very low levels of EGF-induced PI-3 kinase activity relative to cells expressing Gab1. We did observe an approximate 2-fold increase in this low-level basal PI-3 kinase activity in Gab1 -/- MEFs, which represents a Gab1-independent signaling pathway. Gab1 -/- and WT MEFs were additionally stimulated with EGF and the activation of Akt was analyzed by immunoblotting with antibodies which recognize specifically the activated form of Akt. As shown in Figure 1C (top and middle panels), Gab1 -/- cells display no activation of Akt in response to EGF, while WT MEFs show EGF-stimulated Akt activation within two minutes of EGF treatment.

The cDNA encoding the wild type murine Gab1 sequence was cloned into a retroviral vector, and the virus was used to infect Gab1 -/- MEFs. Stable cell lines were selected for co-transduction of a puromycin resistance gene and pools of selected cells were used for further analysis. As shown in Figure 1A, the ectopic Gab1 protein was expressed at slightly lower levels in the Gab1 -/- MEFs relative to endogenous Gab1 expression seen in the wild type MEFs. Quantitation by densitometry reveals Gab1 expression in wild type MEFs to be 1.4-fold higher than ectopic Gab1 expression in Gab1 -/- MEFs. Treatment with EGF induced tyrosine phosphorylation of the exogenous Gab1 protein expressed in the Gab1 -/- MEFs at levels similar to endogenous Gab1 in wild type MEFs. As shown in Figure 1B, expression of exogenous Gab1 in the Gab1 -/- MEFs results in Gab1-associated PI-3 kinase activity that is augmented following EGF treatment. The low level of EGF-induced PI-3 kinase activity observed in the Gab1 -/- cells may be due to signaling via an alternate, Gab1-independent mechanism. These cells were additionally treated with EGF over a period of time and the activation of Akt was assayed by immunoblotting with antibodies specific for the Ser473 phosphorylated form of Akt. The experiment presented in Fig 1C shows that ectopic expression of Gab1 in the Gab1 deficient cells rescues EGF-induced Akt activation to levels similar to those observed in EGF-treated wild type MEFs. Taken together, these results demonstrate that Gab1 is required for EGF-stimulation of PI-3 kinase and Akt.

### The canonical p85 binding sites on Gab1 are essential for PI-3 kinase and Akt activation in response to EGF stimulation

**Figure 2:**
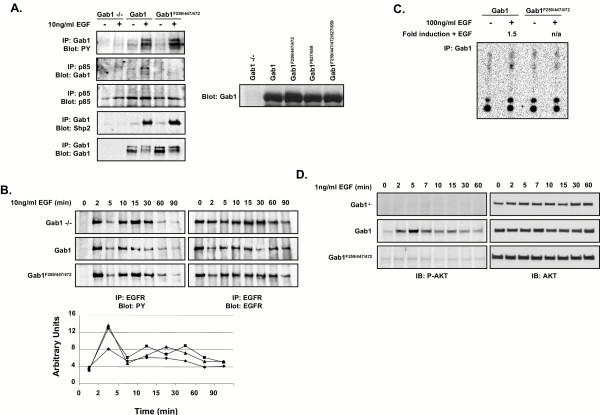
Expression of a Gab1 mutant protein deficient in p85 binding fails to rescue EGF-induced PI-3 kinase/Akt activation in Gab1 deficient MEFs. **A. **The indicated cell lines were serum-starved for 24 hours and stimulated with 10 ng/ml EGF for five minutes at 37°C. Cell extracts were prepared and analyzed for Gab1 tyrosine phosphorylation, Gab1 co-immunoprecipitation with p85, p85 expression, Gab1 co-immunoprecipitation with Shp2, and Gab1 expression. Both Gab1 and Gab1^F446/472/589 ^are tyrosine phosphorylated in response to EGF treatment, and both form a stable complex with Shp2. However, Gab1^F446/472/589 ^fails to associate with the p85 subunit of PI-3 kinase. Additionally total cell lysates from the indicated cell lines were immunoblotted with anti-Gab1 antibodies, providing independent evidence that Gab1 is expressed at approximately equal levels in all cell lines. **B. **The indicated cell lines were serum-starved for 24 hours and stimulated with 10 ng/ml EGF for varying periods of time at 37°C. Cell extracts were prepared and analyzed for EGFR tyrosine phosphorylation. All of the cell lines examined show similar kinetics of EGF-induced EGFR activation. Immunoblots were quantitated by densitometry, normalized for EGFR expression and represented linearly. Diamonds = Gab1 -/-, squares = Gab1, triangles = Gab1^F446/472/589^. **C. **The indicated cell lines were serum-starved for 24 hours and stimulated with 100 ng/ml EGF for five minutes at 37°C. Cell extracts were prepared and Gab1 immunoprecipitates were analyzed for PI-3 kinase activity. Ectopic expression of wild type Gab1 restored EGF-induced PI-3 kinase activity, while expression of Gab1^F446/472/589 ^fails to rescue PI-3 kinase activity in response to EGF treatment. **D. **The indicated cell lines were serum-starved for 24 hours and stimulated with 1 ng/ml EGF for varying periods of time at 37°C. Cell extracts were prepared and analyzed for activation of Akt by using antibodies that specifically recognize the serine473-phosphorylated form of Akt. Membranes were subsequently stripped and immunoblotted for Akt to confirm equal loading. Ectopic expression of Gab1 in Gab1 -/- MEFs rescues activation of Akt in response to EGF treatment, while expression of Gab1^F446/472/589 ^fails to rescue the EGF-induced Akt activation.

The cDNA encoding a mutant Gab1 protein, containing tyrosine to phenylalanine point mutations at the three binding sites for the p85 regulatory subunit of PI-3 kinase (Y446F/Y472F/Y589F) (Gab1^F446/472/589^), was cloned into a retroviral vector and used to generate pools of stable MEF cell lines as described above. We first assayed the cells for Gab1 expression, and for the ability of the mutant Gab1^F446/472/589 ^protein to become tyrosine phosphorylated in response to EGF treatment. As shown in Figure 2A, both wild type Gab1 and Gab1^F446/472/589 ^undergo tyrosine phosphorylation in response to EGF treatment (upper left panel). Quantitation of multiple experiments by densitometry reproducibly demonstrates that Gab1^F446/472/589 ^is tyrosine phosphorylated following EGF treatment to similar levels when normalized for Gab1 expression levels. We next subjected lysates from unstimulated or EGF stimulated cells to immunoprecipitation with anti-Gab1 antibodies followed by immunoblotting with anti-p85 antibodies. As has been demonstrated previously [20], wild type Gab1 readily coimmunoprecipitated p85 following EGF treatment, while the Gab1^F446/472/589 ^mutant protein failed to show an association with p85, confirming that the Gab1^F446/472/589 ^protein is deficient in p85 binding. Similar levels of Gab1 expression in these cells were confirmed by reprobing the Gab1 phosphotyrosine blot with anti-Gab1 antibodies (Figure 2A, bottom left panel). Additionally, total cell lysates of all Gab1 expressing cell lines described in this study were subjected to anti-Gab1 immunoblotting, providing independent evidence for similar levels of Gab1 expression across all cell lines (Figure 2A, right panel).

Because the substrates of Shp2 are for the most part unknown, we were additionally interested in examining the state of EGFR tyrosine phosphorylation following treatment with EGF in order to determine if the failure of Gab1 to bind p85, and potentially recruit Shp2, would influence levels of EGFR autophosphorylation. However, stimulation with EGF for varying time intervals revealed no significant differences in the levels of autophosphorylation of EGFR in cells expressing wild type Gab1 versus the Gab1^F446/472/589 ^mutant (Figure 2B). A linear representation of the EGF-induced EGFR tyrosine phosphorylation following normalization for EGFR expression levels is shown in Figure 2B (bottom). These results are consistent with our finding that p85 binding to Gab1 does not influence the recruitment of Shp2 to the Gab1 signaling complex, and are inconsistent with the conclusion that Gab1 mediates a PI-3 kinase-dependent recruitment of Shp2 [14].

We next explored the role of Gab1 in EGF-induced activation of the PI-3 kinase/Akt cell survival pathway utilizing the Gab1^F446/472/589^expressing cells. We first assayed the Gab1-associated PI-3 kinase activity directly through a PI-3 kinase assay. As shown in Figure 2C, immunoprecipitation of wild type Gab1 following EGF treatment brings down associated PI-3 kinase activity. However immunoprecipitation of Gab1^F446/472/589 ^is not associated with significant levels of PI-3 kinase in the presence or absence of EGF stimulation. In order to assay the effects of EGF stimulation on signaling downstream of PI-3 kinase, Gab1 -/- MEFs expressing no Gab1, wild type Gab1 or Gab1^F446/472/589 ^were treated with EGF over varying times and cell lysates were immunoblotted for serine-phosphorylated Akt. Mutation of the p85 binding sites on Gab1 essentially eliminated all EGF-induced Akt activation relative to cells expressing wild type Gab1 (Figure 2D). The binding of p85 is absolutely required for Gab1-mediated activation of PI-3 kinase and Akt following EGF treatment [9].

Previous work has indicated that treatment of cells with PI-3 kinase inhibitors reduces levels of EGF-induced complex formation between Gab1 and the protein tyrosine phosphatase Shp2 [14]. This finding suggests a role for Gab1 in the PI-3 kinase-dependent recruitment of Shp2 following EGF stimulation. We have examined the possibility that mutation of the p85 binding sites on Gab1, which prevents PI-3 kinase activation, altered EGF-induced recruitment of Shp2 as compared to the recruitment of Shp2 by wild type Gab1. For this purpose, lysates from unstimulated or EGF-stimulated cells were subjected to immunoprecipitation with anti-Gab1 antibodies following immunoblotting with anti-Shp2 antibodies. The experiment presented in Fig 2A shows that mutation of the p85 binding sites on Gab1 did not affect recruitment of Shp2 by Gab1 following EGF stimulation (Figure 2A, fourth panel from the top). As has been previously observed [20], we noted a low level of basal association between Shp2 and both Gab1 and Gab1^F446/472/589^, which may be due to incomplete growth factor starvation prior to EGF stimulation in these experiments.

### Expression of Gab1 mutant protein deficient in Shp2 binding rescues EGF-induced PI-3 kinase/Akt activation in Gab1 -/- MEFs

**Figure 3:**
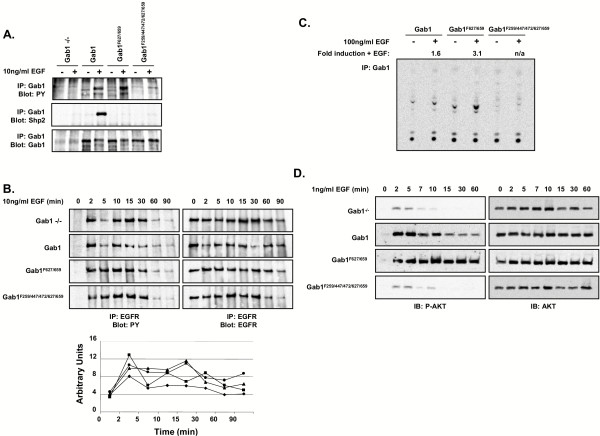
Expression of a Gab1 mutant protein deficient in Shp2 binding enhances EGF-induced activation of the PI-3 kinase/Akt signaling pathway. **A. **The indicated cell lines were serum-starved for 24 hours and stimulated with 10 ng/ml EGF for five minutes at 37°C. Cell extracts were prepared and analyzed for Gab1 tyrosine phosphorylation, Gab1-Shp2 co-immunoprecipitation and Gab1 expression. Gab1^F627/659 ^becomes tyrosine phosphorylated in response to EGF treatment to levels approximately 1.5-fold higher than Gab1 as determined by densitometry, while Gab1^F446/472/589/627/659 ^does not show EGF-induced tyrosine phosphorylation in this assay. Wild type Gab1 forms a stable complex with Shp2 in response to EGF treatment, while Gab1^F627/659 ^and Gab1^F446/472/589/627/659 ^do not. **B. **The indicated cell lines were serum-starved for 24 hours and stimulated with 10 ng/ml EGF for varying periods of time at 37°C. Cell extracts were prepared and analyzed for EGFR tyrosine phosphorylation and EGFR expression. All of the cell lines examined show similar kinetics of EGF-induced EGFR activation. Immunoblots were quantitated by densitometry, normalized for EGFR expression, and represented linearly. Diamonds = Gab1 -/-, squares = Gab1, triangles = Gab1^F627/659^, circles = Gab1^F446/472/589/627/659^. **C. **The indicated cell lines were serum-starved for 24 hours and stimulated with 100 ng/ml EGF for five minutes at 37°C. Cell extracts were prepared and Gab1 immunoprecipitates were analyzed for PI-3 kinase activity. Cells expressing exogenous Gab1^F627/659 ^display enhanced PI-3 kinase activity relative to cells expressing wild type Gab1. Expression of exogenous Gab1^F446/472/589/627/659 ^fails to rescue EGF-induced PI-3 kinase activity in Gab1 deficient MEFs. **D. **The indicated cell lines were serum-starved for 24 hours and stimulated with 1 ng/ml EGF for varying periods of time at 37°C. Cell extracts were prepared and analyzed for activation of Akt by using antibodies that specifically recognize the serine473-phosphorylated form of Akt. Membranes were subsequently stripped and immunoblotted for Akt to confirm equal loading. Cells expressing exogenous Gab1^F627/659 ^display enhanced activation of Akt with sustained kinetics relative to cells expressing wild type Gab1. Expression of exogenous Gab1^F446/472/589/627/659 ^fails to rescue EGF-induced Akt activation in Gab1 deficient MEFs.

To assess the role of the protein tyrosine phosphatase Shp2 in Gab1-mediated signaling induced by EGF, two Gab1 mutants were generated and expressed in pools of Gab1 -/- MEFs. The first contained tyrosine to phenylalanine point mutations at the two binding sites for the Shp2 protein tyrosine phosphatase (Y627F/Y659F) (designated Gab1^F627/659^) and the second contained mutations at the Shp2 binding sites as well as at the three PI-3 kinase binding sites described above (designated Gab1^F446/472/589/627/659^). We first assayed the ability of the mutant Gab1 proteins to become tyrosine phosphorylated in response to EGF. As shown in Figure 3A (top panel) both wild type Gab1 protein and the Gab1^F627/659 ^protein readily undergo tyrosine phosphorylation when stimulated with EGF. Quantitation following densitometry indicates that Gab1^F627/659 ^is reproducibly tyrosine phosphorylated to levels approximately 1.5-fold higher than Gab1. This result suggests that Gab1 may be a substrate of Shp2, and that blocking Shp2 binding thereby increases EGF-induced Gab1 tyrosine phosphorylation. The Gab1^F446/472/589/627/659 ^mutant reproducibly displayed lower levels of tyrosine phosphorylation following treatment with EGF suggesting that these five tyrosines are the main phosphorylation sites on Gab1.

Immunoprecipitation of cell lysates with anti-Gab1 antibodies followed by immunoblotting with anti-Shp2 antibodies demonstrates that wild type Gab1 forms a complex with Shp2 following EGF treatment, while the Gab1^F627/659 ^mutant proteins fail to show an association with Shp2 thus confirming that phosphorylation of Tyr627 and 659 is required for Shp2 binding (Fig 3A). The basal interaction we observed between Gab1 and Shp2 in the absence of EGF stimulation (Figure 2A) is absent in the Gab1^F627/659 ^mutant, even following prolonged exposures of the western blot.

We did not detect a change in the tyrosine phosphorylation of EGFR in cells expressing Gab1 proteins that are deficient in recruitment of Shp2. The experiment presented in Fig 3B shows cells stimulated with EGF over varying periods of time and cell extracts assayed for levels of EGFR tyrosine autophosphorylation. As has been previously reported [15,18,20], recruitment of Shp2 by Gab1 does not alter the magnitude or kinetics of tyrosine autophosphorylation of EGFR (Figure 3B, left panels). Levels of EGFR autophosphorylation are represented linearly following quantitation by densitometry and normalization for protein expression levels (Figure 3B, bottom).

Previous work with Shp2 -/- cells demonstrated an elevated and sustained activation of PI-3 kinase and Akt in response to EGF treatment, and it was proposed that Shp2 may act to dephosphorylate Gab1 at one or both of the p85 binding sites [18]. We utilized the Gab1 proteins deficient in Shp2 binding to assay more directly the role of the Shp2-Gab1 complex in mediating activation of PI-3 kinase and Akt in response to EGF stimulation. As shown in Figure 3C, immunoprecipitation of Gab1^F627/659 ^brings down 1.6-fold higher basal levels of PI-3 kinase activity relative to wild type Gab1 as assayed by PIP3 production. Importantly, Gab1 mutants defective for Shp2 binding show approximately 2-fold higher Gab1-associated PI-3 kinase activity in response to EGF treatment. Consistent with these findings, previous studies have demonstrated that cells transiently over-expressing Gab1^F627/659 ^bound more p85 [18]. In both Gab1 and Gab1^F627/659^expressing cells the Gab1-associated PI-3 kinase activity is augmented by EGF treatment. As expected, the additional mutation of the p85 binding sites eliminates Gab1-associated PI-3 kinase activity. In order to assay the effects of EGF stimulation on signaling downstream of PI-3 kinase, cells were treated with EGF for varying periods of time and cell lysates were assayed for Akt activation by immunoblotting with P-Ser473 Akt antibodies. Interestingly, cells expressing the Gab1^F627/659 ^protein reproducibly showed activation of Akt with significantly sustained kinetics relative to cells expressing wild type Gab1 (Figure 3D, left panels). As expected, the additional mutation of the p85 binding sites (Gab1^F446/472/589/627/659^) limited Akt activation to levels similar to those observed in the Gab1 -/- cells, confirming the requirement for PI-3 kinase association with Gab1 to induce EGF-mediated activation of the Akt pathway. Taken together, these results suggest a role for Shp2 in negatively regulating the EGF induced activation of the PI-3 kinase pathway via Gab1, possibly by dephosphorylating Gab1 at p85 binding sites.

### Expression of ErbB3 in Gab1 -/- MEFs enhances activation of the PI-3 kinase signaling pathway

**Figure 4:**
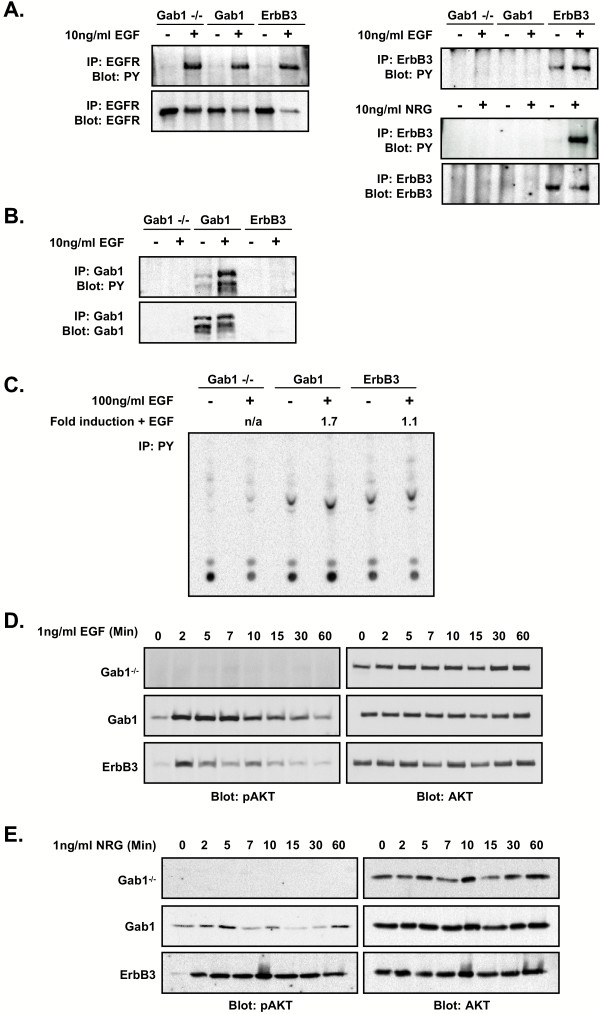
Expression of ErbB3 in Gab1-deficient MEFs partially rescues EGF-induced activation of the PI-3 kinase/Akt signaling pathway. **A. **The indicated cell lines were serum-starved for 24 hours and stimulated with 10 ng/ml EGF for five minutes at 37°C. Cell extracts were analyzed for EGFR tyrosine phosphorylation and EGFR expression, and for ErbB3 tyrosine phosphorylation and ErbB3 expression. Cells were additionally stimulated with 10 ng/ml NRG and cell extracts analyzed for ErbB3 tyrosine phosphorylation. The endogenous EGFR is tyrosine phosphorylated in response to EGF in all cell lines. ErbB3 exhibits weak constitutive tyrosine phosphorylation that is enhanced by NRG treatment, but is not significantly enhanced by treatment with EGF. Selected bands were quantitated by densitometry to determine relative increase in growth factor-induced tyrosine phosphorylation. **B. **The indicated cell lines were serum-starved for 24 hours and stimulated with 10 ng/ml EGF for five minutes at 37°C. Cell extracts were analyzed for Gab1 tyrosine phosphorylation and Gab1 expression. Cells rescued with wild type Gab1 exhibit Gab1 tyrosine phosphorylation in response to EGF treatment, while Gab1 -/- cells and Gab1-deficient cells expressing ErbB3 do not. **C. **The indicated cell lines were serum-starved for 24 hours and stimulated with 100 ng/ml EGF for five minutes at 37°C. Cell extracts were prepared and phosphotyrosine immunoprecipitates were analyzed for PI-3 kinase activity. Expression of wild type Gab1 in Gab1-deficient MEFs rescues the EGF-induced PI-3 kinase activity. Gab1 deficient MEFs exogenously expressing ErbB3 exhibit PI-3 kinase activity that is largely EGF-independent. **D. **The indicated cell lines were serum-starved for 24 hours and stimulated with 1 ng/ml EGF for varying periods of time at 37°. Cell extracts were analyzed for activation of Akt by using antibodies that specifically recognize the phosphorylated form of Akt. Membranes were subsequently stripped and immunoblotted for Akt to confirm equal loading. Ectopic expression of ErbB3 in Gab1 deficient cells results in a partial rescue of EGF-induced Akt activation relative to cells expressing wild type Gab1. **E. **The indicated cell lines were serum-starved for 24 hours and stimulated with 1 ng/ml NRG for varying periods of time at 37°C. Cell extracts were analyzed for activation of Akt by using antibodies that specifically recognize the phosphorylated form of Akt. Membranes were subsequently stripped and immunoblotted for Akt to confirm equal loading. Treatment of Gab1-deficient cells exogenously expressing ErbB3 with NRG results in a robust and sustained activation of Akt, while cells expressing exogenous Gab1 do not exhibit Akt activation in response to NRG treatment.

As described above, PI-3 kinase is recruited to the EGFR via the adaptor protein Gab1. The results presented here demonstrate that Gab1 is required for EGF-induced activation of the PI-3 kinase pathway via the EGFR, presumably because this receptor does not contain binding sites for the p85 regulatory subunit of PI-3 kinase. The catalytically inactive ErbB3 receptor, however, contains at least six binding sites for p85 [5], and thus may bypass the requirement for Gab1 in response to EGF by heterodimerizing with the catalytically active EGFR. In order to test this hypothesis, retroviral vectors were used to introduce either the Gab1 or ErbB3 genes into Gab1 -/- MEFs that endogenously express the EGFR but not ErbB3, and pools of stable cell lines were selected for further analysis. We first assayed the ability of the endogenous EGFR to be tyrosine autophosphorylated in response to EGF, as well as the ability of the exogenous ErbB3 receptor to be tyrosine phosphorylated in response to stimulation with either EGF or neuregulin (NRG). The experiment presented in Fig 4A shows that all cell lines exhibit EGFR autophosphorylation in response to EGF treatment (Figure 4A, upper left panel), while only cells expressing the ectopically introduced ErbB3 protein show ErbB3 tyrosine phosphorylation in response to EGF stimulation. Interestingly, ErbB3 reproducibly shows constitutive low-level tyrosine phosphorylation that is augmented only 1.3-fold in response to EGF treatment. Cells expressing ErbB3 show tyrosine phosphorylation in response to treatment with NRG (Figure 4A, upper and middle right panels). Expression of EGFR and ErbB3 in the appropriate cell lines was confirmed by immunoblotting with antibodies specific for EGFR and ErbB3, respectively (Figure 4A, lower panels). The apparent decrease in EGFR expression in cells co-expressing EGFR and ErbB3 following EGF treatment was not observed in repetitions of this experiment, and is likely due to a stripping anomaly. Additionally, we demonstrated that Gab1 -/- MEFs that express wild type Gab1 display Gab1 tyrosine phosphorylation in response to EGF treatment, while Gab1 -/- control cells or those expressing ErbB3 do not show Gab1 phosphorylation (Figure 4B, upper panel).

In order to test the ability of ErbB3 to rescue the EGF-induced activation of the PI-3 kinase/Akt signaling pathway in Gab1 -/- MEFs, we first assayed these cells for EGF-induced PI-3 kinase activity. Cells were either left unstimulated or were stimulated with EGF and cell lysates were immunoprecipitated with anti-phosphotyrosine antibody. Phosphotyrosine-associated PI-3 kinase activity was then assayed by analysis of PIP3 production. As shown in Figure 4C, both cells expressing wild type Gab1 and ErbB3 show PI-3 kinase activity, while Gab1 -/- cells do not. Interestingly, EGF induces PI-3 kinase activity to a greater degree in cells expressing Gab1 relative to cells expressing ErbB3. In order to assay the effects of EGF stimulation on signaling downstream of PI-3 kinase, cells were treated with EGF over varying periods of time and assayed for the presence of Ser473-phosphorylated Akt. Treatment of cells expressing either wild type Gab1 or ErbB3 with EGF induced rapid activation of Akt, although cells expressing wild type Gab1 reproducibly displaed higher levels of phosphorylated Akt with significantly sustained kinetics relative to Gab1 -/- cells expressing ErbB3 (Figure 4D, left panels). Equal loading and expression levels of Akt were confirmed by immunoblotting (Figure 4D, right panels). Cells expressing ErbB3 displayed activation of Akt in response to treatment with NRG at levels similar to or greater than that seen in Gab1-expressing cells following EGF treatment, with activation reproducibly observed at longer time points (Figure 4E, left panels). Again, equal loading and expression levels of Akt were confirmed by immunoblotting (Figure 4E, right panels). The phosphorylation of AKT in cells expressing Gab1, which shows modest enhancement following treatment with NRG, may be attributed to alternate signaling pathways including those mediated by ErbB2 and ErbB4. Involvement of these receptors was not explored in this study. Taken together, these results demonstrate that ErbB3 can partially compensate for Gab1 deficiency in EGF-induced activation of the PI-3 kinase/Akt signaling pathway, although Gab1-mediated activation appears to be more robust, and likely represents the primary mechanism by which EGF stimulates PI-3 kinase and Akt. While ErbB3 is relatively ineffective at mediating EGF-stimulation of PI-3 kinase activation, it is an efficient mediator of PI-3 kinase stimulation in response to NRG stimulation. Thus, EGF and NRG can stimulate PI-3 kinase activation in normal and transfected cells by means of Gab1 and ErbB3, respectively.

## Conclusions

The results presented here demonstrate an absolute requirement for Gab1 in EGF-induced activation of the PI-3 kinase/Akt signaling pathway. Using this approach we demonstrated a strict requirement for association between Gab1 and p85 in EGF-induced PI-3 kinase activation, suggesting that Gab1 indeed provides an essential link between the EGFR and PI-3 kinase. Additionally, p85 binding does not play a significant role in Shp2 recruitment to the EGFR-Gab1 signaling complex, in contrast to previous studies [14]. We further demonstrated that the Gab1-Shp2 complex is responsible for the negative regulation of the strength and duration of PI-3 kinase/Akt signaling in response to EGF previously observed in Shp2 -/- cells. ErbB3 expression can bypass the requirement for Gab1 in EGFR signaling and can partially rescue EGF-induced activation of the PI-3 kinase/Akt cell survival pathway. This alternate pathway to PI-3 kinase activation may provide cells with a means of controlling either the strength or duration of PI-3 kinase signaling through differential expression of Gab1 and ErbB3, since the ErbB3-mediated response appears to be weaker. Thus Gab1 plays an essential role in bringing together a multi-protein signaling complex in response to EGF that modulates a critical aspect of cellular survival.

## Methods

### Expression constructs

Expression vectors for wild type Gab1 and for Gab1Δp85 (Y446F/Y472F/Y589F) were previously described [21]. The cDNAs of Gab1 (pcDNA3-Gab1-WT), Gab1^F446/472/589 ^(pcDNA3-Gab1-3YF), and ErbB3 (pcDNA3-ErbB3) were subcloned into the mammalian retroviral vector pBabe containing a gene for puromycin resistance. The Gab1^F627/659 ^and Gab1^F446/472/589/627/659 ^mutants were generated by site-directed mutagenesis (Strategene) carried out on pBabe-Gab1-WT (to generate Gab1^F627/659^) or pBabe-Gab1^F446/472/589 ^(to generate Gab1^F446/472/589/627/659^) according to the manufacturers' specifications.

### Cell lines and culture

Gab1-deficient (Gab1 -/-) mouse embryonic fibroblasts (MEFs) were obtained from Walter Birchmeier [22]. Wild type or Gab1-/- MEFs were maintained in DMEM supplemented with 10% fetal bovine serum (Gibco), 2 mM L-glutamine (Gibco), and 100 μg each of penicillin and streptomycin. The retroviral vectors described above (pBabe, pBabe-Gab1, pBabe-Gab1-Gab1^F446/472/589^, pBabe-Gab1-Gab1^F627/659^, pBabe-Gab1-Gab1^F446/472/589/627/659^, and pBabe-ErbB3) were used to transfect the amphitropic retroviral packaging cell line GPG (obtained from Joan Brugge) and high-titer viral stocks were used to infect Gab1 -/- MEFs. Cells were selected in medium supplemented with puromycin and pools of selected cells were used for further experiments. Prior to stimulation with EGF, cells were starved in serum-free medium.

### Immunoprecipitation and immunoblotting

Cells were grown on 15-cm plates as described above to approximately 80% confluence and then starved overnight in DMEM without serum. Cells were left unstimulated or were stimulated with recombinant human EGF (Invitrogen) as indicated. Stimulations were halted by the addition of ice-cold PBS. Cells were washed in PBS and lysed in buffer containing 1% Triton X-100 as previously described [23]. For Gab1 immunoprecipitations, a mixture of polyclonal antibodies directed against both the N- and C-termini of Gab1 and cross-linked to Protein A Sepharose (Zymed) was incubated with cell extracts for 2–4 hours at 4°C. For p85 immunoprecipitations, a mixture of polyclonal antibodies directed against the N-terminal SH2 domain of p85 and the full-length p85 protein (Upstate) was incubated with cell extracts for 2–4 hours at 4°C. For EGFR immunoprecipitations, a polyclonal antibody directed against the C-terminus of the EGFR was incubated with cell extracts overnight at 4°C. For ErbB3 immunoprecipitations, a mixture of polyclonal antibodies directed against both the N- and C-termini of ErbB3 was incubated with cell extracts overnight at 4°C. Protein A Sepharose was added to immunoprecipitates (except anti-Gab1) and incubated for 1 hour at 4°C. Immunoprecipitates were then washed in buffer containing 0.1% Triton X-100, separated by SDS/PAGE, and transferred to nitrocellulose membranes (Bio-Rad). For Akt immunoblotting, total cell extracts were separated directly by SDS/PAGE and transferred to nitrocellulose membranes. Membranes were blocked for 1 hour or overnight in 5% BSA/TBS and immunoblotted as indicated. The anti-phosphotyrosine blotting was carried out with antibody 4G10 (Upstate). Anti-Gab1, anti-EGFR and anti-ErbB3 blotting was performed with the indicated polyclonal antibodies (Upstate). Anti-Shp2 blotting was carried out with polyclonal antibody (Santa Cruz). Anti-phosphoSer473-Akt and anti-Akt blotting were performed with the respective polyclonal antibodies (Cell Signaling Technology). Proteins were visualized by incubation with Enhanced Chemiluminescence (Amersham Pharmacia) according to the manufacturer's specifications.

### PI-3 kinase assay

The PI-3 kinase assay was performed essentially as previously described [22-24]. Briefly, cells were serum-starved for 24 hours and stimulated with EGF as indicated, and cell extracts were prepared as described above. The lysates were immunoprecipitated using anti-Gab1 polyclonal antibodies or the monoclonal anti-phosphotyrosine antibody PY20 (Santa Cruz) for 2 hours at 4°C. Protein A Sepharose was incubated with immunoprecipitates for 1 hour at 4°C. The immunoprecipitates were washed three times with Buffer 1 (1X PBS, 1% NP-40), twice with Buffer 2 (0.5 M LiCl, 0.1 M Tris pH 7.5), twice with TNE (10 mM Tris pH 7.5, 100 mM NaCl, 1 mM EDTA pH 8.0), and twice with Buffer 4 (20 mM Hepes pH 7.5, 50 mM NaCl, 5 mM EDTA pH 8.0, 0.03% NP-40, 30 mM Tatrasodium Pyrophosphate (Sigma)). L-α-Phosphatidylinositol (Sigma) was added (10 μl of a sonicated solution at 10 mg/ml in 20 mM HEPES pH 7.5) and the reaction was initiated by the addition of 50 μl of the kinase buffer (20 mM Tris pH 7.5, 75 mM NaCl, 10 mM MgCl_2 _10 μM ATP, 100 mM adenosine, 10 μCi [γ-^32^P] ATP) per sample. Samples were incubated at 30°C for 15 minutes, and the reaction was stopped by addition of 100 μl 1N HCl. Samples were extracted by addition of 200 μl CHCl_3_/CH_3_OH (1:1). The samples were vortexed and centrifuged, and the lower organic phases containing phospholipids were dried at 27°C for two hours. Samples were resuspended in 10 μl of PI-4-P standard (0.5 ml CHCl_3_, 0.5 ml CH_3_OH, 2.5 μl HCl, 1 mg L-α-Phosphatidylinositol 4-monophosphate (Sigma)) and subjected to thin layer chromatography (TLC plates – VWR) in CHCl_3_/CH_3_OH/NH_4_OH/H_2_O (45:35:7:3). The TLC plates were exposed in a PhosphorImager cassette for four days.

## Authors' contributions

BL carried on the initial biochemical experiments before leaving the laboratory. DM continued this study and carried out the biochemical studies, the PI-3 kinase assays, and drafted the manuscript. IL participated in the PI-3 kinase assays. JS initiated and supervised the project. All authors read and approved the final manuscript.
